# P02-09 The dissemination of an effective school-based PA intervention programme: Sigue la Huella (Follow the footprint)

**DOI:** 10.1093/eurpub/ckac095.028

**Published:** 2022-08-29

**Authors:** Hisham Bohouri, Sonia Asun, José Antonio Julián, Eduardo Ibor, Lena Lhuisset, Nicolas Fabre, Katrien De Cocker, Caera Grady, Enrique García Bengoechea

**Affiliations:** 1 University of Zaragoza, Huesca, Spain; 2 Université de Pau et des Pays de l'Adour, Tarbes, France; 3 University of Ghent, Ghent, Belgium; 4 University of Limerick, Limerick, Ireland

**Keywords:** school-based interventions, physical activity, dissemination, adolescents

## Abstract

**Background:**

A widespread dissemination of effective evidence-based physical activity (PA) interventions is needed whether a greater proportion of the population, who could potentially benefit from it, wants to be reached (Finch et al., 2016). ‘Sigue la Huella' (Follow the Footprint), is one of the few effective evidence-informed PA school-based interventions conducted on adolescents in Europe, with the support of the family and the community (Murillo et al., 2014). The main aim of this study is to describe and analyze the process of dissemination of this intervention program.

**Methods:**

The ‘Sigue la Huella' was implemented at one secondary school situated in Jaca/Huesca (Spain). The Replicating Effective Programs (REP) framework was used, because provides a roadmap for disseminating effective interventions (Kilbourne et al., 2007). The intervention was delivered through workshops, ongoing technical assistance to the teachers, and the distribution of an instructional guide among the teachers and the school staff. A quasi-experimental design was adopted to examine the effect of ‘Sigue la Huella' after its dissemination process. PA was assessed using accelerometers at baseline and after the intervention of 14 weeks. In addition, we evaluated the dissemination process using the PRACTIS guide (Koorts et al., 2018).

**Results:**

From an initial evaluation of the dissemination process, several key learnings emerged:

- To identify strategies for effective stakeholders engagement;
- To overcome some barriers such as the excess workload of the teachers in charge of disseminating the intervention program;
- For better measures to determine the success of dissemination approaches (e.g., reliable and valid indicators of organizational and policy change).

**Conclusions:**

This study provides a comprehensive overview of the dissemination process of an effective school-based program identifying contextual barriers and facilitators that influence program implementation in new contexts and the direction of specific strategies to address those barriers.

